# Book Notices

**Published:** 1868-06-15

**Authors:** 


### *BOOK NOTICES.*

---

MATERIA MEDICA, for the Use of Students. By John B. Biddle, M.D., Professor of Materia Medica in the Jefferson Medical College, etc., etc. Third edition, enlarged. With illustrations. Philadelphia: Lindsay & Blakiston. 1868. Pp. 384. W. B. Keen & Co., 148 Lake St., Chicago.

This is a thoroughly revised and enlarged edition of Prof. Biddle's work on Materia Medica. It is designed to present the leading facts and principles usually comprised under this head, as set forth by the standard authorities, and to fill a vacuum which seems to exist in the want of an elementary work on the subject. The larger works usually recommended as Text Books in our Medical Schools are too voluminous for convenient use. This work will be found to contain, in a condensed form, all that is most valuable, and will supply students with a reliable guide to the courses of lectures on Materia Medica, as delivered at the various Medical Schools in the United States.

---

CONTRIBUTIONS RELATING TO THE CAUSATION AND PREVENTION OF DISEASE, AND TO CAMP DISEASES; together with a report of the diseases, etc., among the prisoners at Andersonville,

Ga. Edited by Austin Flint, M.D. New York: Published for the U. S. Sanitary Commission, by Hurd & Houghton, 459 Broome St. 1867. Pp. 667.

Prof. Flint, in this compilation, has discharged his editorial duties with his usual ability and fidelity. We shall take great pleasure, so soon as the pressure on our columns will admit, in commenting on this very useful work. Meanwhile, we commend it to the careful perusal of our readers.

---

The American Journal of Obstetrics, and Diseases of Women and Children. Edited by E. Noeggerath, M.D., Physician to the German Hospital and Dispensary, and B. F. Dawson, M.D., Lecturer on Uterine Pathology in Medical Department of the University of New York. $3.00 a Year, in advance; Single copies, $1.00. Moorhead, Bond & Co., Publishers, No. 60 Duane St., N. Y.

The first number of this Journal was issued on May 15th, 1868, and it will be published quarterly thereafter. It consists of 96 pages, elegantly printed, and contains Original Articles, Reports of Societies, Hospitals, Lectures, and a complete review of Foreign and Domestic Literature of the above subjects. Articles for the Journal, and subscriptions, are solicited, which, together with all communications, are to be addressed to the Publishers.

The initial No. has been received, and fully sustains our expectations. The Essay by Dr. Jacobi, on the Pathology and Treatment of Croup, alone is worth more than the annual subscription price.

